# Voice-Based Screening of Depression and Anxiety Using Machine Learning and Deep Learning: A Scoping Review of Methods and Clinical Readiness

**DOI:** 10.3390/life16091467

**Published:** 2026-09-02

**Authors:** Mădălina Muntean-Codrea, Bogdan Nemeș, Horia George Coman, Ciprian Ionuț Băcilă, Raluca Nicoleta Trifu, Radu Flaviu Oroian, Marinela Minodora Manea, Dana Cristina Herța, Sergiu Șușman

**Affiliations:** 1 Department of Medical Psychology and Psychiatry, Iuliu Hațieganu University of Medicine and Pharmacy, 400012 Cluj-Napoca, Romania; madalina.codrea@gmail.com (M.M.-C.); hcoman@umfcluj.ro (H.G.C.); raluca.trifu@umfcluj.ro (R.N.T.); mmanea@umfcluj.ro (M.M.M.); dherta@umfcluj.ro (D.C.H.); 2 Faculty of Medicine, Lucian Blaga University of Sibiu, 550024 Sibiu, Romania; ciprian.bacila@ulbsibiu.ro; 3 Zalău County Emergency Hospital, 450129 Zalău, Romania; radu.oroian@gmail.com; 4 Department of Histology, Iuliu Hațieganu University of Medicine and Pharmacy, 400012 Cluj-Napoca, Romania; histologiedidactic@elearn.umfcluj.ro

**Keywords:** depressive disorder, anxiety disorders, machine learning, deep learning, speech analysis, voice biomarkers, clinical readiness, scoping review

## Abstract

Despite the high global prevalence of depressive and anxiety disorders, access to early clinical assessment remains limited for many. Although this field has grown rapidly, existing reviews have focused primarily on technical performance, with limited systematic attention to whether current models meet the prerequisites for clinical implementation. This scoping review mapped methodological approaches across this domain and evaluated clinical readiness using five predefined indicators: sample size adequacy, external validation, prospective data collection, real-world evaluation, and model explainability. Searches of PubMed, Web of Science, and IEEE Xplore (March 2026) identified 2463 records; 34 studies (37 dataset evaluations) were included. The majority of studies (91%) were published from 2022 onwards. Depression was the primary target in 91% of studies, while only one study addressed anxiety. Hand-crafted acoustic features were the most frequent (57%), while classical machine learning was the most common model type (32%). External validation was conducted in only 32% of evaluations and real-world testing in 8%. Clinical readiness was classified as Low in 24%, Moderate in 65%, and High in 11% of evaluations. No evaluation met all five indicators simultaneously. These findings apply primarily to voice-based depression screening; 36 of 37 evaluations targeted depression, and the evidence base for anxiety disorders is limited to a single evaluation, precluding comparable characterisation for that condition. The principal challenges to clinical implementation are insufficient external validation, reliance on laboratory conditions, narrow linguistic coverage, and inconsistent metric reporting. The framework applied in this scoping review provides a replicable structure for assessing the clinical validity of AI-driven psychiatric screening tools.

## 1. Introduction

Depressive and anxiety disorders represent some of the most common and debilitating mental health conditions worldwide. The World Health Organisation estimates that depression affects 280 million people globally, making it one of the leading causes of disability, while anxiety disorders affect a further 301 million across all age groups [1,2]. In routine clinical practice, the assessment of depression and anxiety relies on structured diagnostic interviews, standardised clinician-administered scales and self-report instruments used for population screening. While these approaches remain the gold standard for diagnosis, they are resource-intensive, require trained clinicians, and are subject to inter-rater variability and reporting biases [3]. The identification of objective, quantifiable biomarkers capable of augmenting or supporting clinical assessment has therefore attracted considerable research interest [4].

Voice and speech have emerged as promising biomarkers for affective disorders, with their neurobiological basis well established [4]. Depressive states are associated with dysregulation of cortico-limbic circuits governing motor control and emotional regulation, producing psychomotor changes that are reflected in speech [4]. This results in characteristic alterations in acoustic speech parameters, including reduced fundamental frequency variability, diminished vocal energy, slowed speech rate, increased pause duration, impaired articulation, and a flattened prosodic contour [4]. Systematic quantification of these features through digital signal processing and machine learning enables detection with greater objectivity, consistency, and scalability than clinical observation alone [5].

Research on AI-based voice screening for depression and anxiety has grown rapidly, yet no agreement exists on how studies should be designed, reported, or evaluated [5]. The field integrates clinical psychiatry, speech and language processing, biomedical engineering and digital health, disciplines that rarely share the same reporting conventions or quality standards, making synthesis difficult to conduct.

Systematic reviews and meta-analyses addressing diagnostic performance already exist [6,7]; however, a meta-analytic approach is not suited to the specific question of clinical readiness, which cannot be quantitatively pooled and requires structured appraisal across multiple translational dimensions such as dataset adequacy, validation design, real-world evaluation, and model interpretability, areas that existing performance-focused syntheses have not addressed. A scoping review enables comprehensive mapping of the field without the restrictive eligibility criteria of a systematic review. It is well-suited to identifying methodological trends, research gaps, and barriers to clinical translation.

Several reviews have examined this field, each contributing to the evidence base while revealing its limitations [5]. conducted a foundational systematic review of voice biomarkers for psychiatric disorders. The study found that machine learning models showed promising performance but identified substantial methodological challenges, calling for standardised study design guidelines. More recent work has reached similar conclusions. A 2025 meta-analysis in JMIR Mental Health, which covered 105 studies published between 2013 and 2025, quantified diagnostic performance using pooled sensitivity and specificity. Despite this, the authors concluded that clinical readiness remains limited, citing small sample sizes, overfitting risks, and the absence of real-world validation [8]. A 2025 review published in the Journal of Voice reflected these concerns, flagging that promising accuracy figures are offset by methodological heterogeneity and poor generalisability. The authors recommended standardised protocols, more diverse datasets, and real-world testing before clinical adoption can be considered [6].

Existing reviews have predominantly characterised technical performance and identified methodological challenges, with limited systematic attention to translational prerequisites for clinical adoption. While these reviews noted that clinical readiness remains limited and called for larger samples and real-world validation, none have applied a structured, multi-indicator appraisal framework to formally map clinical readiness across the field [5,6,8].

This review is motivated by the gap between algorithmic progress and clinical readiness. Although classification performance has improved, it is unclear whether these gains represent true translational progress or are due to methodological artefacts, overfitting, or evaluation practices that do not reflect clinical conditions. By applying a predefined clinical readiness framework and mapping methodological approaches, this review provides a structured and replicable basis for assessing the translational maturity of current evidence.

The primary objective of this scoping review was to map the machine learning and deep learning approaches used for voice-based screening of depressive and anxiety disorders, while evaluating the extent to which these models demonstrate readiness for clinical implementation. Clinical readiness was assessed using five predefined indicators derived from established clinical prediction model evaluation standards and AI deployment frameworks [7,9]: sample size adequacy, external validation, prospective data collection, real-world or clinical setting evaluation, and model explainability.

This review identified 34 studies published between 2018 and 2026, contributing 37 qualifying dataset evaluations. Clinical readiness was classified as Low in 24%, Moderate in 65%, and High in 11% of evaluations, with no evaluation meeting all five readiness indicators simultaneously. The principal barriers to clinical implementation were insufficient external validation, reliance on controlled laboratory conditions, narrow linguistic coverage, and inconsistent performance reporting.

## 2. Materials and Methods

*Study Design.* This study was conducted as a scoping review following the methodological framework proposed by Arksey and O’Malley [10] and refined by Levac et al. [11] and the Joanna Briggs Institute (JBI) [12]. Reporting adhered to the Preferred Reporting Items for Systematic Reviews and Meta-Analyses Extension for Scoping Reviews (PRISMA-ScR) checklist [13]. The review protocol was registered post hoc on the Open Science Framework (OSF) (https://doi.org/10.17605/OSF.IO/2VZGF). The literature search was conducted on 23 March 2026, before registration. Registration was completed retrospectively to ensure methodological transparency.

The primary objective of this scoping review was to map the machine learning and deep learning approaches used for voice-based screening of depressive and anxiety disorders and evaluate the extent to which these models demonstrate readiness for clinical implementation. Secondary objectives were to: (1) identify commonly used acoustic features and speech representations; (2) examine model architectures and analytical techniques; (3) characterise datasets and study populations; (4) evaluate validation strategies employed across studies; (5) assess the use of explainable artificial intelligence methods; (6) identify methodological limitations and barriers to clinical adoption; and (7) highlight gaps that may inform future research directions.

*Eligibility Criteria.* Eligibility criteria were defined using the Population–Concept–Context (PCC) framework.

*Population.* Studies involving adolescents or adults evaluated for depressive disorders and/or anxiety disorders were considered eligible, irrespective of clinical setting, age group, or country of data collection. Healthy volunteers serving as control participants were also eligible.

*Concept.* The review focused on studies that applied machine learning or deep learning techniques to voice or speech audio data for screening, detection, classification, or risk assessment of depressive or anxiety disorders. For the purpose of this review, classification was defined as the identification or prediction of depression and/or anxiety status as a distinct binary or multi-class target outcome, and did not include general emotion recognition or affective state classification unrelated to these clinical conditions.

*Context.* No restrictions were placed on study setting. Clinical, community, telemedicine, and research environments were all considered eligible, reflecting the broad range of contexts in which this technology is being developed and evaluated.

*Inclusion Criteria.* Studies were included if they met all the following criteria: (1) original empirical research published as a peer-reviewed journal article or conference proceeding; (2) use of human participants (adolescents or adults); (3) use of clinician-administered diagnostic interviews or validated clinical scales administered by a clinician as ground truth labels for depression and/or anxiety, with depression and/or anxiety defined as a distinct target outcome; (4) use of speech or voice audio as the sole modality for model development and evaluation; (5) total sample size of at least 50 participants with at least 25 in the target group; (6) application of a clearly described machine learning or deep learning model; (7) subject-level data separation at model evaluation; (8) reporting of quantitative classification performance metrics including at least one of accuracy, F1-score, AUC, sensitivity, or specificity; and (9) publication in English from January 2015 onward.

*Exclusion Criteria.* Studies were excluded if they met any of the following criteria: (1) non-original research (reviews, protocols, editorials, conference abstracts without full empirical results); (2) non-human or synthetic data; (3) non-clinical or insufficiently defined populations, including studies using exclusively self-administered symptom scales as diagnostic labels; (4) target condition mismatch (generic emotion recognition, affective state detection, or studies not reporting depression or anxiety results separately); (5) multimodal designs combining speech with any additional modality; (6) text-only analyses; (7) insufficient sample size; (8) absence of a clearly described machine learning or deep learning model; (9) segment-level data splitting without subject-level separation at final evaluation; (10) absence of quantitative performance metrics or insufficient methodological detail to allow interpretation.

A post hoc clarification of the labelling validity criterion was applied during the full-text review. Studies were included only if they demonstrated at least one dataset with a clinician-administered diagnostic assessment. Studies relying exclusively on self-administered instruments, including widely used benchmark datasets labelled PHQ-8, PHQ-9, or BDI-II, without clinician involvement, were excluded due to concerns about construct validity [14,15]. Where a study used both a self-report-labelled and a clinician-labelled dataset, it was retained, but results from the self-report-labelled dataset were not included in the primary synthesis.

*Information Sources.* A systematic search was conducted across three electronic databases: PubMed/MEDLINE, Web of Science, and IEEE Xplore. These databases were selected to ensure comprehensive coverage of biomedical and engineering literature relevant to the review topic. The search was executed on 23 March 2026. Reference lists of included studies were not screened for additional sources, consistent with the review scope.

*Search Strategy.* Search strategies were developed using controlled vocabulary and free-text terms related to voice and speech analysis, machine learning and deep learning, and depressive and anxiety disorders. The full search strategies for each database were as follows.

PubMed/MEDLINE:

((“Voice”[Mesh] OR “Speech”[Mesh] OR voice[tiab] OR speech[tiab]

OR vocal*[tiab] OR acoustic*[tiab] OR prosod*[tiab])

AND

(“Machine Learning”[Mesh] OR “Artificial Intelligence”[Mesh]

OR “Deep Learning”[Mesh] OR machine learning[tiab]

OR deep learning[tiab] OR artificial intelligence[tiab]

OR neural network*[tiab] OR algorithm*[tiab])

AND

(“Depressive Disorder”[Mesh] OR “Depression”[Mesh]

OR “Anxiety Disorders”[Mesh] OR depression[tiab]

OR depressive[tiab] OR anxiety[tiab]))

Filters applied: English language; publication date from January 2015.

Web of Science:

TS = ((voice OR speech OR vocal* OR acoustic* OR prosod*)

AND

(“machine learning” OR “deep learning”

OR “artificial intelligence” OR “neural network*”

OR algorithm*)

AND

(depression OR “depressive disorder*”

OR anxiety OR “anxiety disorder*”))

Filters applied: English language; publication date from January 2015; document types restricted to articles.

IEEE Xplore:

((“All Metadata”:voice OR “All Metadata”:speech

OR “All Metadata”:acoustic OR “All Metadata”:vocal)

AND

(“All Metadata”:“machine learning”

OR “All Metadata”:“deep learning”

OR “All Metadata”:“artificial intelligence”

OR “All Metadata”:“neural network”)

AND

(“All Metadata”:depression

OR “All Metadata”:“depressive disorder”

OR “All Metadata”:anxiety))

Filters applied: publication date from January 2015.

**Figure 1:**
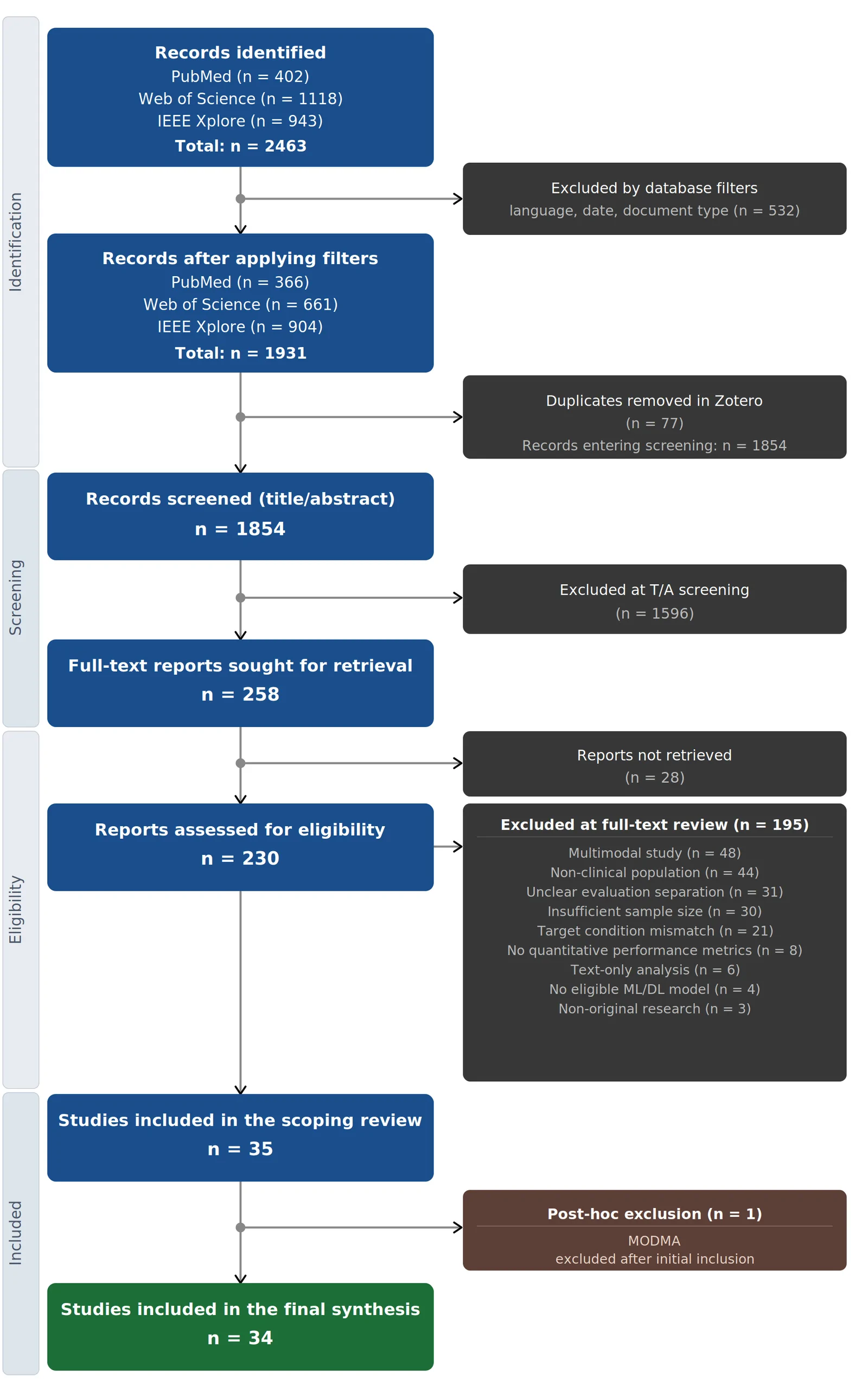
PRISMA-ScR flow diagram.

*Selection of Sources of Evidence.* All records identified through database searches were exported to Zotero (version 7.0.9, 64-bit) for reference management throughout the review. Duplicate removal was performed within Zotero before screening. Title and abstract screening and full-text eligibility assessment were conducted independently by two reviewers using a structured eligibility checklist based on inclusion and exclusion criteria. Disagreements were resolved by discussion. Reasons for exclusion at the full-text stage were recorded for each excluded record. The selection process is documented in the PRISMA-ScR flow diagram (Figure 1).

*Data Charting Process.* Data were charted for all included studies [16,17,18,19,20,21,22,23,24,25,26,27,28,29,30,31,32,33,34,35,36,37,38,39,40,41,42,43,44,45,46,47,48,49,50] independently by two reviewers using a standardised extraction form developed and pilot-tested prior to full data collection. The charting form was implemented in Microsoft Excel. The following variables were extracted for each included study: author(s) and year of publication; country of data collection; study design; sample size and population characteristics including age and clinical setting; target disorder; dataset identity and type; voice recording conditions including environment and device; speech task type; acoustic features or embeddings used; machine learning or deep learning model type and architecture; use of pre-trained models; validation approach and data split strategy; quantitative performance metrics; use of explainable artificial intelligence methods and specific methods employed; and variables required for clinical readiness appraisal. Reported limitations were also extracted, as documented by the study authors.

When a single study reported results across multiple datasets, each dataset was evaluated as a separate row to enable dataset-level synthesis. Assumptions and simplifications made during extraction were documented within the charting form. Discrepancies between reviewers were resolved by discussion.

*Data Items.* The extracted data items are listed in full in the charting form (Supplementary Table S1). Key variables included acoustic feature category (hand-crafted, deep embeddings, end-to-end, or mixed), model architecture category (classical machine learning, CNN, CNN + RNN variants, transformer, ensemble, HMM/GMM, or hybrid), data split strategy (subject-level, segment-level, or mixed), external validation status, prospective data collection status, real-world evaluation status, and model explainability status. Performance metrics were extracted as reported by study authors without standardisation or transformation.

*Critical Appraisal and Clinical Readiness Framework.* Consistent with scoping review methodology, a formal risk-of-bias assessment was not conducted. A structured methodological appraisal was performed to contextualise the translational maturity of the evidence base, using a clinical readiness framework developed a priori in accordance with established clinical prediction model evaluation standards and AI deployment frameworks [7,9], and specified in the review protocol. Each qualifying dataset evaluation was assessed against five predefined binary indicators:

(1) Sample size adequacy: categorised as below 100, 100–1000, or above 1000 total participants per dataset evaluation, reflecting the statistical reliability of performance estimates. A sample below 100 was considered insufficient for stable performance estimation across most classification architectures, regardless of other design features. The threshold of 1000 identifies evaluations with substantially greater statistical reliability than the field median observed in this review (median *N* = 118), while the 100–1000 range represents the majority of the evidence base. These thresholds were selected as pragmatic stratification points broadly consistent with minimum sample size considerations for clinical prediction model development [7], and do not account for class distribution, model complexity, predictor dimensionality, validation strategy, or the precision required for performance estimates; they should be interpreted as approximate stratification criteria rather than absolute adequacy standards.
(2) External validation: whether a model trained on one dataset was evaluated on a second, independently collected dataset with no overlap in participants, collection site, or collection period (present/absent), reflecting generalisability beyond the training context. Cross-validation partitions and held-out subsets from the same dataset do not satisfy this criterion.
(3) Prospective data collection: whether speech recordings and clinical assessments were obtained concurrently within a prospectively designed study protocol, rather than derived retrospectively from archival sources. This indicator concerns the design of the data-generating study, not the model pipeline, and is distinct from prospective model validation, the deployment of a pre-specified model to accruing cases prior to outcome ascertainment, which no evaluation in this review achieved.
(4) Real-world or clinical setting evaluation: whether the evaluation was conducted in an active clinical or community setting rather than a controlled research protocol (present/absent), reflecting deployment feasibility.
(5) Model explainability: whether interpretable modelling approaches or post hoc explainable artificial intelligence methods were applied and reported (present/absent), reflecting the transparency requirements of clinical decision support tools.

Based on the profile of these indicators, each dataset evaluation was categorised as follows: Low readiness: no external validation, sample size below 100, and no real-world evaluation; Moderate readiness: at least one of the following present: external validation, sample size of 100 or above, or real-world evaluation; High readiness: external validation present plus at least one of the following: sample size above 1000, prospective data collection, or real-world clinical evaluation. The three categories are mutually exclusive and hierarchically ordered: an evaluation satisfying High readiness criteria necessarily also satisfies Moderate criteria, but is classified at the higher level. An evaluation with external validation present but no sample exceeding 1000, no prospective data collection, and no real-world evaluation would be classified as Moderate, since it satisfies at least one Moderate criterion but does not meet the additional threshold required for High classification.

The composite classification was designed to reflect the most consequential translational prerequisites rather than to aggregate all five indicators uniformly, in accordance with established clinical prediction model evaluation standards and AI deployment frameworks [7,9]. External validation and real-world evaluation were treated as the primary qualifying criteria for higher readiness categories because they directly address generalisability and deployment feasibility, the two conditions without which clinical implementation cannot be considered. Model explainability was operationalised as a parallel indicator: its presence or absence was recorded for each evaluation and is visible in Supplementary Table S2, but it was not incorporated as a sequential prerequisite within the composite classification, given that an unexplainable model lacking external validation cannot be considered deployment-ready regardless of its interpretability. Sample size was applied as a floor criterion distinguishing evaluations with fundamentally insufficient statistical power from those meeting a minimum threshold for reliable performance estimation. The five indicators are presented both as individual binary profiles in Supplementary Table S2 and as a composite classification, enabling readers to identify specific readiness gaps independently of the summary category. Although formal risk-of-bias assessment is outside the scope of scoping review methodology, publication bias cannot be excluded: studies reporting higher classification performance are more likely to be published, which may inflate the apparent performance figures summarised in this review.

*Synthesis of Results.* Extracted data were synthesised descriptively using summary tables and narrative summaries. Findings were organised thematically according to the review’s secondary objectives, covering acoustic feature extraction approaches, model architectures, dataset characteristics, validation practices, explainability, and indicators of translational readiness. A meta-analysis was not performed due to anticipated and confirmed heterogeneity across study designs, datasets, and outcome measures. Descriptive statistics, including frequencies and proportions, were computed for all categorical variables. Performance metrics were summarised as ranges and medians where sufficient data were available, and heterogeneity in metric reporting was documented as a substantive finding

## 3. Results

### 3.1. Study Selection

The database search retrieved 2463 records across PubMed (*N* = 402), Web of Science (n = 1118), and IEEE Xplore (n = 943). After applying database-level filters for language, publication date, and document type, 1931 records remained. Duplicate removal was performed within Zotero, eliminating 77 records and yielding 1854 records for screening. Title and abstract screening excluded a further 1596 records. The remaining 258 full-text reports were sought for retrieval; 28 could not be retrieved. The remaining 230 reports were assessed for eligibility; 195 were excluded, most frequently for being multimodal studies (n = 48) or for involving non-clinical or insufficiently defined populations (n = 44), including studies that relied exclusively on self-administered labelling instruments. Thirty-five studies initially meeting all eligibility criteria were included, contributing 38 qualifying dataset evaluations. Following a comprehensive re-examination of data splitting practices prompted by peer review (see Section 3.7), one evaluation [17], (MODMA dataset) was excluded post hoc after confirmation of segment-level splitting without subject-level separation at final evaluation, violating inclusion criterion 9. The final synthesis therefore includes 34 studies and 37 qualifying dataset evaluations. The selection process is summarised in the PRISMA-ScR flow diagram (Figure 1).

### 3.2. Study Characteristics

The 34 included studies were published between 2018 and 2026. Publication volume increased markedly over the review period, with 31 studies (91%) appearing from 2022 onwards and 15 studies (44%) published in 2025 or 2026 alone. The majority were journal articles (n = 27, 79%), with seven conference proceedings (21%). The predominant study design was cross-sectional (n = 20, 59%), followed by experimental studies using pre-existing datasets (n = 10, 29%) and three prospective longitudinal cohort studies (n = 3, 9%). One study was retrospective in design.

Data collection spanned 12 countries or regions. China was the most represented data collection context, appearing in 19 of the 34 studies, followed by the United States (n = 13). European sites contributed to seven studies, with Italy (n = 3), Germany (n = 2), the Netherlands (n = 1), and Turkey (n = 1). Additional contributing regions included South Korea (n = 2), Japan (n = 2), Brazil (n = 1), and India (n = 1).

Target disorders. Depression was the primary focus of 31 studies (91%). Two studies targeted both depression and another disorder (bipolar disorder or suicide risk within depressive disorder), and one study exclusively examined anxiety (TRAILS cohort, The Netherlands).

### 3.3. Datasets and Study Populations

Across the 37 qualifying dataset evaluations, total sample sizes ranged from 50 to 7959 participants, with a median of 118. Thirteen evaluations (35%) used samples smaller than 100 participants, 21 (57%) fell in the 100 to 1000 range, and three (8%) exceeded 1000 participants, these being the CONVERGE corpus (n = 7959, appearing in two studies) and a large multi-centre Chinese case–control dataset (n = 1808).

The most frequently used dataset was MODMA (n = 5 evaluations), a publicly available Mandarin-language corpus of clinically diagnosed patients, followed by CMDC (n = 4), the Androids corpus (n = 4), and CONVERGE (n = 2). Twelve evaluations (32%) used privately collected datasets not available for independent replication. Public datasets accounted for 25 of 37 evaluations (68%), though this figure requires contextualisation: several nominally public datasets carried labelling approaches that did not meet the clinical assessment threshold of this review and were therefore excluded from synthesis, most notably DAIC-WOZ and the AVEC corpora.

A critical and recurring finding was the linguistic concentration of included evaluations. Chinese-language datasets accounted for 23 of 37 evaluations (62%), with Italian (n = 4, 11%), Korean (n = 2, 5%), and Japanese (n = 2, 5%) each contributing modestly. German, Turkish, Portuguese, and Dutch datasets each appeared in one or two evaluations. This concentration reflects the availability of pre-existing clinician-labelled corpora rather than the global burden of depressive and anxiety disorders, and represents a significant constraint on the generalisability of current models.

### 3.4. Speech Tasks and Recording Conditions

Speech elicitation paradigms varied considerably across studies. Mixed task designs, combining two or more task types within the same evaluation, were most prevalent (n = 13, 35%), followed by structured clinical interviews (n = 11, 30%), read speech (n = 10, 27%), and spontaneous speech (n = 3, 8%). The dominance of structured interviews and read speech tasks reflects the constraints of clinical data-collection contexts, where naturalistic, continuous recording is rarely feasible. Only three evaluations used spontaneous speech collected outside a structured protocol.

Recording environments were predominantly controlled laboratory or clinical settings. Three evaluations used naturalistic recording conditions: two involved telephone-based clinical interviews, and one used participants’ own smartphones to collect WhatsApp voice messages. This near-exclusive reliance on controlled conditions is a recognised barrier to deployment in real-world clinical workflows.

### 3.5. Acoustic Features and Speech Representations

Hand-crafted acoustic features remained the dominant representation strategy, used in 21 of 37 evaluations (58%). These included Mel-frequency cepstral coefficients (MFCCs), filter bank energies, prosodic features (fundamental frequency, jitter, shimmer, energy), formants, and large-scale feature sets extracted via openSMILE, including eGeMAPS, ComParE, and emoLarge configurations. Deep embeddings derived from pre-trained models, including wav2vec 2.0, x-vectors, and audio-spectrogram transformers, were used in 8 evaluations (22%). Mixed approaches combining hand-crafted and deep features were used in six evaluations (16%), and purely end-to-end representations, in which raw waveforms were passed directly to learnable filters, were used in two evaluations (5%).

Pre-trained models were used in a substantial proportion of studies. Transfer learning from speech recognition (wav2vec 2.0), speaker verification (x-vector, TDNN), audio classification (VGGish, YAMNet, AudioSet), and speech emotion recognition corpora (CREMA-D, RAVDESS) was employed across multiple architectures. This trend reflects the field’s growing adoption of foundation models trained on large-scale, general-purpose corpora, adapted for depression or anxiety classification through fine-tuning.

### 3.6. Model Architectures

Classical machine learning classifiers constituted the largest single model category, appearing in 12 evaluations (32%). These included support vector machines, random forests, logistic regression, XGBoost, and linear discriminant analysis, often applied to large hand-crafted feature vectors with dimensionality reduction. Convolutional neural network variants combined with recurrent components (CNN + LSTM, CNN + BiGRU, CNN + TDNN) were used in eight evaluations (22%), while standalone CNN architectures were used in six (16%). Transformer-based models appeared in four evaluations (11%), and ensemble approaches, including stacking, mixture-of-experts, and multi-task ensemble frameworks, in four (11%). Hybrid architectures combining convolutional, transformer, and recurrent components were employed in two evaluations (5%). One evaluation used a classical HMM-GMM generative framework.

The co-existence of classical and deep learning approaches reflects the heterogeneity of dataset sizes in the field. Classical ML classifiers were disproportionately represented in evaluations with smaller samples (below 100 or in the lower 100–1000 range), where the data volume is insufficient to reliably train deep architectures. Transformer models appeared exclusively in evaluations using publicly available or larger private datasets.

### 3.7. Validation Strategies and Data Split Practices

Subject-level partitioning, ensuring that no participant’s data appeared in more than one training, validation, or test partition, was applied in all 37 qualifying evaluations. In 32 evaluations (86%), this partitioning was also the unit of model training and inference: models were trained and evaluated directly on subject-exclusive partitions. In the remaining five evaluations (14%), speech segments, rather than whole-subject recordings, were used as the input unit for model training and inference within each subject-exclusive partition, and segment-level predictions were subsequently aggregated by majority voting into a single subject-level classification for evaluation. In these five evaluations, subject-exclusivity was enforced at the partitioning stage, prior to model fitting, and majority voting served solely as a prediction-aggregation step rather than as a substitute for partition-level separation; accordingly, no participant’s data contributed to both model training and evaluation, and these evaluations satisfied inclusion criterion 7 without risk of speaker-dependent data leakage.

External validation, evaluation of a trained model on an independent dataset not used during model development, was present in 12 of 37 evaluations (32%). In the majority of cases (n = 25, 68%), models were evaluated exclusively on held-out portions of the same dataset used for training, thereby limiting the evidence available to support generalisation claims. The strength of evidence within this category was not uniform: three evaluations [23,35,37], applied a pre-specified model to a prospectively collected, fully independent clinical cohort, representing the most rigorous form of external validation; the remaining nine evaluations involved transfer of a fixed trained model to a second independently collected corpus, which provides a weaker basis for generalisability claims. Prospective data collection was present in 14 evaluations (38%). Real-world or clinical deployment evaluation, testing in an active clinical or community setting, rather than a controlled research protocol, was documented in only three evaluations (8%).

### 3.8. Performance Metrics and Reporting Heterogeneity

Performance reporting was heterogeneous across evaluations, precluding quantitative synthesis or cross-study comparison. AUC was reported in 16 of 37 evaluations (43%), with values ranging from 0.659 to 1.000 and a median of 0.861. F1-score was reported in 25 evaluations (68%), ranging from 0.589 to 0.992 with a median of 0.856. Five evaluations (14%) reported neither AUC nor F1-score, relying solely on accuracy, sensitivity, and specificity, or on regression metrics such as RMSE and MAE. The high median values observed for both AUC and F1 must be interpreted cautiously, given that a substantial proportion of evaluations were conducted on small, imbalanced datasets without external validation, conditions that systematically inflate apparent performance.

Accuracy was reported inconsistently and is difficult to interpret without class distribution information. Several evaluations on datasets skewed toward healthy controls reported high accuracy, primarily reflecting the correct identification of the majority class. The lack of a field-wide standard metric is itself a methodological barrier to evidence synthesis and clinical translation, a point returned to in the discussion.

### 3.9. Explainable Artificial Intelligence

Explainability methods were present in 16 of 37 evaluations (43%). Methods included feature importance ranking (via SHAP, spFSR, Pearson correlation, Gini importance, and random forest importance scores), attention visualisation (TFCA attention maps, t-SNE feature distribution plots), post hoc attribution methods (SHAP on Mel spectrograms, LIME), clinically grounded feature design with explicit symptom mapping, and architecture-intrinsic interpretability (HMM state path monitoring, symptom-level multitask prediction). SHAP was the most commonly used post hoc method, appearing in four evaluations.

The 21 evaluations (57%) without any explainability component represent a notable gap, particularly given that interpretability is widely cited as a prerequisite for clinical adoption of algorithmic diagnostic support tools [9].

### 3.10. Clinical Readiness Appraisal

Clinical readiness was assessed across five predefined indicators derived from established clinical prediction model evaluation standards and AI deployment frameworks [7,9]: sample size adequacy, external validation, prospective data collection, real-world or clinical deployment evaluation, and model explainability. Results are presented in Supplementary Table S2.

Distribution of readiness categories. Nine evaluations (24%) were classified as Low readiness, 24 (65%) as Moderate, and four (11%) as High. No evaluation met all five indicators simultaneously.

Low-readiness evaluations were characterised by small sample sizes (all below 100 participants), absence of external validation, and absence of real-world evaluation. Most involved secondary evaluations of the CMDC or MODMA corpus or small, private datasets conducted in controlled laboratory conditions.

Moderate readiness evaluations constituted the majority of the evidence base. The most common route to Moderate classification was based solely on sample-size adequacy (12 evaluations with N in the 100–1000 range, without external or real-world validation). A further eight Moderate evaluations also demonstrated prospective data collection or external validation, representing a more substantive evidence base.

High readiness evaluations were identified in four evaluations across three studies. Lin et al. [35] achieved the strongest overall profile by combining external validation in an independent clinical cohort, prospective data collection, real-world evaluation via smartphone recordings in outpatient and home settings, and model explainability via SHAP. Xu et al. [23] demonstrated external validation across two independent, prospectively collected datasets, with explainability. Li et al. [37] achieved High readiness across two dataset evaluations through external validation, a prospective design, and clinically grounded feature interpretability in a paediatric population.

Indicators driving readiness gaps. Across all 37 evaluations, the most consistently absent indicator was real-world or clinical deployment evaluation (present in 8%), followed by external validation (present in 32%). Prospective data collection was absent in 62% of evaluations. These patterns indicate that the dominant limitation is not algorithmic performance but the absence of evaluation designs capable of supporting translation claims. The concentration of evaluations under controlled laboratory conditions, on small, homogeneous datasets without independent replication, collectively defines the current ceiling of clinical readiness in this field.

## 4. Discussion

### 4.1. Summary of Evidence Against the Primary Objective

This scoping review mapped machine learning and deep learning approaches applied to voice-based screening of depressive and anxiety disorders across 34 studies and 37 qualifying dataset evaluations published between 2018 and 2026. The central finding is that the technical capacity to distinguish clinically diagnosed individuals from healthy controls using speech acoustics is well established [5,8], but the evidence base supporting clinical implementation remains underdeveloped. Only four of 37 qualifying dataset evaluations (11%) met criteria for High clinical readiness, and no evaluation satisfied all five predefined indicators simultaneously. The majority, 24 evaluations (65%), occupied a Moderate readiness category characterised by adequate sample sizes but absent external validation and no real-world evaluation. These readiness characterisations apply specifically to voice-based depression screening; 36 of 37 included evaluations targeted depression, and the single anxiety-focused evaluation [46] precludes any parallel readiness conclusions for that condition. This pattern indicates the field has advanced substantially at the algorithmic level, while translational infrastructure, prospective design, independent replication, and clinical deployment testing have lagged considerably behind.

The rapid acceleration in publication volume, with 91% of included studies appearing from 2022 onwards, confirms that voice-based depression screening is an active and growing research domain. The adoption of deep learning architectures, pre-trained foundation models, and transformer-based representations reflects the broader trajectory of speech and audio processing. However, acceleration in methodological sophistication has not been matched by maturation in evaluation rigour. The dominance of retrospective experimental designs (29% of studies), near-exclusive reliance on controlled laboratory recording conditions, and concentration of evaluations on a small number of publicly available corpora together limit conclusions about how these models would perform in clinical practice.

The single anxiety-focused study [46] and the two studies addressing comorbid or compound presentations highlight a pronounced gap in the evidence base for anxiety disorders. The near-exclusive focus on depression reflects the availability of labelled corpora rather than clinical priority, and represents a research direction that remains largely unexplored in the voice-based screening literature.

### 4.2. Methodological Limitations and Barriers to Clinical Adoption

Dataset characteristics and labelling validity. A recurrent and fundamental issue across the literature, and a primary driver of exclusions in this review, is the conflation of self-administered symptom severity scales with clinical diagnosis. Instruments such as the PHQ-8, PHQ-9, and BDI-II were designed as screening and monitoring tools, not diagnostic instruments [14,15], and their use as ground-truth labels for binary classification introduces construct validity problems rarely acknowledged in engineering-oriented publications. This review operationalised clinical labelling validity as a hard inclusion criterion, requiring clinician-administered diagnostic interviews or validated structured instruments administered by trained clinicians. Applying this criterion excluded a substantial portion of otherwise eligible literature, including several widely cited benchmark datasets. The implication is significant: performance figures reported on self-report-labelled datasets cannot be straightforwardly interpreted as evidence of clinical diagnostic accuracy.

Sample size and statistical power. Thirty-five percent of qualifying dataset evaluations involved fewer than 100 participants, and the median total sample size was 118. These figures are modest relative to the complexity of the classification task and the number of model parameters typically optimised. Small samples increase the risk of overfitting, reduce the reliability of cross-validation estimates, and make reported performance metrics highly sensitive to the specific train-test partition used [7]. The three evaluations exceeding 1000 participants, two using the CONVERGE corpus and one large Chinese multi-centre case–control dataset, represent outliers in a field where sample acquisition is constrained by logistical and ethical demands of clinical data collection.

Evaluation, separation and data leakage. Data split strategy was an explicit eligibility criterion in this review, and segment-level splitting, in which audio segments from the same speaker appear in both the training and evaluation sets, was a primary exclusion criterion (n = 32 evaluations excluded, including one excluded post hoc following re-examination of the source publication). Among included studies, 86% applied subject-level separation throughout, which is the minimum standard for a valid generalisation estimate in speaker-dependent tasks. However, even subject-level cross-validation on a single dataset does not constitute external validation, and the distinction between these two forms of evaluation is frequently blurred in the literature. The high reported performance figures in several studies that used cross-validation on small, single-site datasets should be interpreted with this caveat in mind.

External validation and generalisability. External validation, testing a trained model on an independent dataset collected in a different context, population, or time period, was absent in 68% of qualifying evaluations. This is the single most consequential gap identified in this review. A model trained and evaluated on one dataset, regardless of apparent performance, provides no direct evidence of generalisability to a new clinical population, recording device, language, or healthcare system [7]. The one qualifying evaluation that did conduct a genuine cross-dataset, cross-lingual evaluation observed substantial performance degradation, underscoring the sensitivity of acoustic depression models to domain shift [24]. For a screening tool to be clinically deployable, it must demonstrate stable performance across the demographic and contextual diversity encountered in routine care, a standard that the current evidence base does not meet.

Recording conditions and ecological validity. Controlled laboratory recording conditions dominated the evidence base, with only three evaluations conducted in naturalistic or real-world settings. Clinical deployment of voice-based screening tools would require robust performance under conditions substantially different from those used in model development: variable ambient noise, diverse recording devices including consumer smartphones, spontaneous conversational speech rather than structured tasks, and speakers with comorbidities, medication effects, and age-related voice changes. The ecological validity gap between laboratory performance and anticipated deployment conditions is a barrier that has received insufficient systematic attention in the field.

Linguistic and demographic generalisability. The concentration of qualifying evaluations in Chinese-language datasets (62%) reflects the historical availability of clinician-labelled corpora in China and the productive research infrastructure developed around them. While this concentration has accelerated progress within that linguistic context, it limits the generalisability of current models to other languages, dialects, and cultural contexts. Acoustic correlates of depression, including prosodic patterns, speech rate norms, and articulation characteristics, are known to vary across languages and cultures [16]. Models trained exclusively on Mandarin-speaking clinical populations cannot be assumed to generalise to Arabic, Hindi, Swahili, or even European-language speakers without revalidation. The near-absence of African, Middle Eastern, and South Asian language representations in the evidence base is a critical equity gap, given that the global burden of depressive disorders is disproportionately concentrated in low- and middle-income countries [1,2].

Performance metric heterogeneity. The absence of a standardised reporting framework across the field makes cross-study comparison and meta-analytic synthesis difficult and unreliable. AUC, arguably the most clinically interpretable metric for a screening tool, as it captures the trade-off between sensitivity and specificity across decision thresholds, was reported in only 43% of evaluations. F1-score, which combines precision and recall into a single value without distinguishing their individual clinical implications, was the most commonly reported metric. Accuracy without accompanying class-distribution information is of limited interpretability in imbalanced datasets and should not be considered sufficient as a standalone performance metric. The absence of confidence intervals, calibration assessments, and decision-threshold analyses in most studies further limits the clinical meaningfulness of the reported figures. The TRIPOD statement and its extension for AI-based prediction models [7,18] provide established reporting standards that, if adopted field-wide, would substantially improve comparability of evidence and support more rigorous evaluation of clinical utility.

Explainability and clinical trust. Explainability methods were absent in 57% of qualifying evaluations. For a voice-based screening tool to be integrated into clinical practice, clinicians must understand which acoustic signal properties drive model predictions, verify that these properties are clinically plausible, and identify when predictions may be unreliable. Black-box classifiers, even those with high reported performance, face significant barriers to adoption in regulated clinical environments [9]. The studies that incorporated explainability methods demonstrated that acoustic features associated with model predictions can be mapped onto clinically recognised symptoms such as reduced vocal energy, slowed speech rate, and monotone prosody, lending face validity to the models and supporting clinician engagement. The minority status of explainability in the current evidence base represents a missed opportunity to bridge the gap between algorithmic performance and clinical credibility.

### 4.3. Gaps and Future Research Directions

Prospective, multi-site clinical studies. The most consequential gap in the current evidence base is the near-absence of prospective studies designed from the outset for clinical translation. The four High-readiness evaluations identified in this review, drawn from three studies [23,35,37], share a common feature: they were designed with clinical deployment in mind, incorporating prospective data collection, independent test sets, and evaluation in settings approximating real-world use. Future studies should prioritise prospective cohort designs with pre-registered protocols, multi-site data collection to capture demographic and contextual diversity, and independent validation cohorts defined prior to model training.

Standardised evaluation benchmarks with clinician-administered labels. The field would benefit substantially from the development and open release of large-scale, multi-language, clinician-labelled speech corpora analogous in scale to CONVERGE but with rigorous diagnostic protocols. The CONVERGE corpus, with its CIDI-based diagnostic interviews and sample exceeding 7000 participants, represents a model for what is needed, but its restriction to Chinese-speaking women limits its generalisability. International collaborative initiatives to develop comparable corpora in under-represented languages should be prioritised. Equally important is the establishment of agreed-upon benchmark evaluation protocols that specify subject-level splitting, required test set sizes, and mandatory metric reporting, including AUC, sensitivity, specificity, and calibration.

Anxiety disorders. The evidence base for voice-based anxiety screening is effectively absent, with a single included study addressing social anxiety disorder in adolescents. Given the high prevalence, functional burden, and treatment gap associated with anxiety disorders globally, and for which a theoretical basis in hyperarousal, altered breathing, and voice tremor has been proposed, this represents a major opportunity for targeted research. Future work should address generalised anxiety disorder, social anxiety disorder, and panic disorder as distinct targets, rather than treating anxiety as a secondary or comorbid outcome.

Ecological validity and device-agnostic models. Future studies should systematically evaluate model performance under ecologically valid conditions, including naturalistic recording environments, consumer-grade devices, and conversational speech. Domain adaptation and noise-robust training strategies should be incorporated into model development pipelines from the outset rather than as post hoc additions. Studies using smartphones as the primary recording device, as demonstrated by Lin et al. [35] and Otani et al. [31], represent a promising direction for accessible, scalable screening tools that can be deployed in primary care or community settings.

Demographic and linguistic diversity. Future research should explicitly address the linguistic and demographic gaps identified in this review. This includes the development of models and corpora for African, South Asian, and Middle Eastern language groups; age-stratified models addressing adolescent and older adult populations, for whom the acoustic correlates of depression may differ from working-age adults; and sex-disaggregated analyses that go beyond reporting combined performance to examining differential model behaviour across demographic subgroups.

Longitudinal and treatment-response designs. The three prospective longitudinal studies included in this review [42,46,47] represent a methodologically advanced class of designs that move beyond cross-sectional classification toward tracking symptom trajectories and treatment response. Voice-based monitoring of treatment response, relapse prediction, and early warning signal detection are clinically compelling applications that require longitudinal data and repeated-measures evaluation designs. Future investment in this direction would extend the utility of voice-based models beyond point-in-time screening.

Standardised explainability frameworks. The field lacks consensus on what constitutes adequate model explainability for clinical deployment. Future work should move beyond post hoc feature-importance ranking, which explains individual predictions but does not establish mechanistic validity, toward clinically grounded interpretability frameworks that connect model behaviour to the neurobiological and psychophysiological mechanisms underlying depression and anxiety. Collaboration among speech scientists, clinical psychologists, and psychiatrists in the design and evaluation of explainability outputs would accelerate the development of clinician-facing interfaces that support rather than replace clinical judgement.

### 4.4. Limitations of This Review

Several limitations of this scoping review should be acknowledged. First, the search was conducted across three databases, PubMed, Web of Science, and IEEE Xplore, which, while representing the primary repositories for biomedical and engineering literature, may not capture all relevant work in this domain. Sources not covered include Scopus, ACM Digital Library, arXiv (sections cs.SD, eess.AS, and cs.LG), and the ISCA archive, which indexes INTERSPEECH proceedings comprehensively. Grey literature, preprint servers, and backward or forward citation snowballing were also outside the scope of this review. These omissions may have resulted in underrepresentation of ML/DL conference work indexed variably across the databases searched. Additionally, although the English-language restriction was applied as a pragmatic eligibility criterion, its interaction with the linguistic concentration finding warrants acknowledgement: the datasets underlying the 63% figure, MODMA, CMDC, CONVERGE, and the Androids corpus, are publicly available corpora documented in English and constitute the standard reference base of the field internationally, but whether inclusion of Chinese-language publications would have altered this proportion cannot be determined without additional searches in Chinese-language databases such as CNKI or Wanfang.

Second, although screening and data charting were conducted independently by two reviewers and discrepancies were resolved through discussion, no inter-rater agreement statistic was calculated or recorded, and the individual pre-reconciliation forms are no longer available. The absence of a quantified agreement metric is acknowledged as a limitation, particularly for readiness indicators requiring interpretive judgement, such as real-world evaluation and prospective data collection. Extraction errors cannot be entirely ruled out, particularly for complex methodological variables such as data-splitting strategies and clinical assessment instruments; where ambiguity was encountered in the primary study reporting, conservative interpretations were applied.

Third, the clinical readiness appraisal framework applied in this review was developed a priori based on established clinical prediction model evaluation standards and AI deployment frameworks [9,18], but has not been independently validated as a composite instrument. While each of the five indicators is individually grounded in the literature, alternative weighting schemes or indicator sets could produce different readiness classifications. The framework should therefore be interpreted as a structured organising tool for evidence mapping rather than a formal quality assessment instrument. The sample size thresholds applied in the clinical readiness framework (<100, 100–1000, >1000) were defined pragmatically and do not incorporate class imbalance ratios, model parameter counts, predictor dimensionality, validation strategy, or the statistical precision required for reliable performance estimation. The binary coding of external validation does not distinguish between evaluation designs that range from assessment in a prospectively collected independent clinical cohort to transfer of a fixed trained model across independently collected benchmarks; this heterogeneity limits the strength of conclusions attributable to the ‘present’ category.

Fourth, the rapidly evolving nature of the field means that studies published after the search date, including preprints and in-press articles, are not captured in this review. Given that 91% of included studies were published from 2022 onwards, the evidence base is likely to have continued to expand since the search was conducted.

Fifth, the focus on binary or multi-class classification of depression and anxiety status as the primary outcome excluded a substantial body of work on severity estimation, treatment monitoring, and dimensional symptom prediction. While this boundary was necessary to maintain a tractable, clinically coherent review scope, it means the review does not capture the full landscape of voice-based computational psychiatry.

## Data Availability

No new data were created or analysed in this study. The charting form and extracted dataset used to support the findings of this review are available from the corresponding author upon reasonable request.

## Supplementary Materials

The following supporting information can be downloaded at: https://www.mdpi.com/article/10.3390/life16091467/s1, Table S1: Study Characteristics (full); Table S2: Clinical Readiness Appraisal.

## Abbreviations

The following abbreviations are used in this manuscript:

ACM	Association for Computing Machinery
AI	Artificial Intelligence
AUC	Area Under the Receiver Operating Characteristic Curve
AVEC	Audio/Visual Emotion Challenge
BDI-II	Beck Depression Inventory-II
BiGRU	Bidirectional Gated Recurrent Unit
CIDI	Composite International Diagnostic Interview
CMDC	Chinese Multi-centre Depression Corpus
CNKI	China National Knowledge Infrastructure
CNN	Convolutional Neural Network
ComParE	Computational Paralinguistics Challenge Feature Set
CONVERGE	China, Oxford, and Virginia Commonwealth University Experimental Research on Genetic Epidemiology
CREMA-D	Crowd-sourced Emotional Multimodal Actors Dataset
DAIC-WOZ	Distress Analysis Interview Corpus-Wizard of Oz
DL	Deep Learning
eGeMAPS	Extended Geneva Minimalistic Acoustic Parameter Set
GMM	Gaussian Mixture Model
HMM	Hidden Markov Model
IEEE	Institute of Electrical and Electronics Engineers
ISCA	International Speech Communication Association
JBI	Joanna Briggs Institute
LIME	Local Interpretable Model-agnostic Explanations
LSTM	Long Short-Term Memory
MAE	Mean Absolute Error
MEDLINE	Medical Literature Analysis and Retrieval System Online
MFCC	Mel-Frequency Cepstral Coefficients
ML	Machine Learning
MODMA	Multimodal Open Dataset for Mental Disorder Analysis
OSF	Open Science Framework
PCC	Population–Concept–Context
PHQ-8	Patient Health Questionnaire-8
PHQ-9	Patient Health Questionnaire-9
PRISMA	Preferred Reporting Items for Systematic Reviews and Meta-Analyses
PRISMA-ScR	PRISMA Extension for Scoping Reviews
RAVDESS	Ryerson Audio-Visual Database of Emotional Speech and Song
RMSE	Root Mean Square Error
RNN	Recurrent Neural Network
SHAP	SHapley Additive exPlanations
SVM	Support Vector Machine
TDNN	Time Delay Neural Network
TRAILS	TRacking Adolescents’ Individual Lives Survey
TRIPOD	Transparent Reporting of a Multivariable Prediction Model for Individual Prognosis or Diagnosis
t-SNE	t-distributed Stochastic Neighbour Embedding
XAI	Explainable Artificial Intelligence
XGBoost	Extreme Gradient Boosting
